# Women’s experience of the decision-making process for home-based postnatal midwifery care when discharged early from hospital: A Swedish interview study

**DOI:** 10.18332/ejm/152547

**Published:** 2022-09-09

**Authors:** Margareta Johansson, Li Thies-Lagergren

**Affiliations:** 1 Department of Women’s and Children’s Health, Uppsala University Hospital, Uppsala University, Uppsala, Sweden; 2 Department of Midwifery Research, Reproductive, Perinatal and Sexual Health, Department of Health Sciences, Faculty of Medicine, Lund University, Lund, Sweden

**Keywords:** decision-making, experiences, home-based care, midwifery care, mothers, postnatal care

## Abstract

**INTRODUCTION:**

Women and their families are often excluded from reproductive decision-making processes in postnatal care, and do not know which choices they have. Shared decision-making is a critical but challenging component of maternity care quality. The aim was to explore women’s experience of the decision-making process about early return from hospital with home-based postnatal midwifery care.

**METHODS:**

This is a descriptive qualitative study. In total, 24 women participated in a semi-structured telephone interview, averaging 58 minutes. Data were analyzed using thematic analysis according to Braun and Clarke.

**RESULTS:**

The main theme explored was ‘The supremacy of giving new mothers autonomy to decide on the postnatal care model they would prefer’. Important aspects of the women’s decision-making process were the time-point for receiving information about the home-based midwifery model of care, to receive sufficient time for consideration about the model, to have a rationale for choosing home-based care, and to comprehend the concept.

**CONCLUSIONS:**

Women must be given sufficient time for consideration and necessary information about postnatal care models, which is essential for making an informed decision. Parents’ readiness for discharge must be identified by midwives who need to facilitate shared decision-making by introducing early postnatal care model choices, describe these options, and support women to explore their preferences. Midwives must ensure parents’ participation in decision-making for the time of discharge from hospital.

## INTRODUCTION

Women and their families are often excluded from reproductive decision-making processes in postnatal care, and do not know which choices they have^1,2^. A postnatal care model decision should be influenced by exploring and respecting what matters most to women as individuals. This exploration in turn depends on developed informed preferences as new mothers^3^. A randomized trial found that women who received home-based care reported fewer problems with breastfeeding and greater satisfaction with the help given, compared to women who received hospital-based postnatal care^4^. Postnatal care in hospitals is generally experienced to be a busy place with inadequate numbers of caregivers with few opportunities for women to rest and recover after childbirth^5^. The length of the hospital stay following birth has decreased over time^6^, likely due to economic reasons^7^ or shortage of hospital beds^8^. Early discharge has been defined as returning home within 24–48 hours after birth^9^. Nevertheless, there has been no correlation reported between length of stay and overall satisfaction with postnatal care^6,7^. Not all women might be ready to be discharged early thus wishing a longer stay, even when the birth had been uncomplicated and they are physically healthy^10^. Women are most likely to have a positive experience of an early discharge if they have been prepared for it, have a partner and access to family, and support by a midwife^9^. Women are increasingly expected to be involved in decisions made during the postpartum period^1^, and to be allowed flexibility in decision-making, thus embracing a more personalized model of postnatal care to augment the decision-making process^11^. There is a lack of understanding if women are introduced to postnatal care choices, if options of care are described, and if they are supported to explore their own postnatal care preferences^12^. The aim of this study was to explore women’s experience of the decision-making process about early discharge from hospital with home-based postnatal midwifery care.

## METHODS

### Study design and setting

This study involved a descriptive qualitative design. Postnatal care may be provided at a hospital, in a patient hotel/family suite or at home with or without organization of home-based midwifery care. Currently in Sweden, there is a lack of national postnatal care guidelines and postnatal care differs from hospital to hospital. In some hospitals only the mother and the baby are allowed to stay, in others both parents can stay. After an uncomplicated birth, women are most often recommended early discharge without home-based midwifery care^13^.

A Swedish home-based postnatal midwifery care model was implemented in September 2015 and was offered to those families meeting the criteria for early discharge (6–24 hours after birth). The day after postnatal discharge, a midwife contacts the family for support via telephone to plan follow-up consisting of: 1) daily telephone contact, 2) home visits, and 3) hospital visits; as preferred and needed during the first week after childbirth. The women could also use a 24-hour hotline to the hospital if they need additional support^14,15^.

### Participants

Inclusion criteria were: be a Swedish-speaking mother, have had an uncomplicated pregnancy and a vaginal birth with no complications, a healthy term baby, had participated in the home-based postnatal midwifery care, and had answered ‘yes’ regarding participation in an in-depth interview study in a web-based follow-up survey. During an on-going 12-month period, 180 women completed the survey. Of these, 69 women indicated that they could be contacted for further follow-up. Potential participants were contacted one to three months after birth of the baby. A total of 35 women were contacted via email/short text messages to confirm interest in participation in an in-depth interview and 24 women accepted to participate in a telephone interview. During the last interviews, there was data repetition and a decision was made to terminate the data collection. Data collection was carried out for 5–18 weeks postpartum (on average 11 weeks).

Background characteristics of the included women (Table 1), were identified in the online quantitative survey. Identified variables were age, marital status, education level, number of children, time of hospital discharge after birth of their baby (6–12 hours; >12–24 hours), if discharge was regarded at the right time (Yes; No, I had been able to go home earlier; No, I needed a longer stay), and preferred model of postnatal care for next child (home-based, hotel-based, traditional hospital-based, do not know).

**Table 1:** Background characteristics for the study participants, (N=24)

*Characteristics*	*n (%)*
**Age** (years)	
28–41	mean age 34
**Marital status**	
Married/cohabiting	24 (100)
Other	0 (0)
**Education level**	
High school	3 (12.5)
College/University	21 (87.5)
**Number of children**	
1	1 (4.2)
2–4	23 (95.8)
**Time of hospital discharge after birth** (hours)	
6–12	16 (66.7)
>12–24	8 (33.3)
**Experienced discharge at the right time** (as preferred)	
Yes	22 (91.7)
No, I had been able to go home earlier	1 (4.2)
No, I needed a longer stay	1 (4.2)
**Preferred model of postnatal care for next child**	
Home-based care	20 (83.3)
Hotel-based care	2 (8.3)
Traditional hospital-based care	1 (4.2)
Do not know	1 (4.2)

### Measures and variables

Qualitative semi-structured audio-recorded telephone interviews were conducted between April and September 2017, at a time and day preferred by the participant. The interviews followed an interview guide (Table 2). The length of the interviews varied between 27 and 114 minutes (58 minutes on average). The interviews also included aspects of their overall experiences of receiving home-based postnatal care and these will be reported elsewhere.

**Table 2:** Included questions in the interview guide

How was the decision-making process to receive home-based postnatal midwifery care experienced?
What did you know about different options of postnatal care models?
What did you know about the possibility to receive home-based postnatal midwifery care?
Personally, what do you think about differences, if any, between postnatal care at home compared to a hospital?

### Data analysis

The data were analyzed using thematic analysis according to Braun and Clarke^16^. First, the authors familiarized themselves with the verbatim transcribed data, reading and re-reading the data, and noting initial ideas. Initial codes with relevant features according to the aim and collating data relevant to each code were then generated. Thereafter, codes were sorted into seven potential themes: making a decision of care model, receiving information about home-based postnatal care, have had an uncomplicated childbirth, woman and baby in good health, post-birth follow-up, prepared for motherhood, and to be in the home or at the hospital environment. The initial potential themes were reviewed and condensed into four new themes. The main theme, ‘The supremacy of giving new mothers autonomy to decide on the postnatal care model they would prefer’, was explored which described the women’s experience of the decision-making process for home-based postnatal midwifery care (Table 3). The last phase in the data analysis was to include a selection of vivid, compelling quotes in the findings of the study^16^.

**Table 3:** The main theme and its explanatory themes

*The supremacy of giving new mothers autonomy to decide on the postnatal care model they would prefer:*
Time-point for receiving information is relevant for the decision-making process
Time for consideration about postnatal care choices is asked for
The rationale for postnatal care model choice
To comprehend the concept of postnatal care models facilitates decision-making

### Ethical considerations

Participation in the study was voluntary and informed consent was collected from all participants before the interviews started. All participating women were informed about confidentiality and that they could withdraw their participation at any time. All participants talked freely in a friendly atmosphere, and no one withdrew their participation. The current study has received ethical approval from the Ethics Committee in Stockholm (dnr: 2016/1163-32/1).

## RESULTS

The analysis generated the main theme: ‘The supremacy of giving new mothers autonomy to decide on the postnatal care model they would prefer’, which describes how the women experienced the decision-making process, and is explained by four themes (Table 3).

### Time-point for receiving postnatal care information is relevant for the decision-making process

The included women described having received information about the hospital’s model of home-based postnatal midwifery care, either during pregnancy at the antenatal care unit or after birth at the maternity unit. Several women considered it very important to receive information about postnatal care models available, already during pregnancy. This would imply that the expectant parents could think about it, could talk it through, could plan it and be able to present their preference themselves after the birth of their baby, which generated a sense of parental security. For the women who had received information about the model of home-based postnatal midwifery care during pregnancy, the decision-process of choosing this model of care had eventually emerged. A second-time mother emphasized the importance of receiving prenatal information about the home-based postnatal model in detail and said:

*‘Then you become even more familiar with what it [home-based care] is and what it implies; that you are admitted [at the hospital] and getting the same care as at the hospital.’* (Participant 14)

A few women described that the midwife did not give information to them about the home-based postnatal midwifery care after the birth of their baby. Since the information was provided during pregnancy, the women asked for this care model themselves. A second-time mother said:

*‘I requested this model of care and was offered [midwifery] care at home.’* (Participant 2)

Several women described that they had not received any information about the home-based postnatal midwifery care during pregnancy, so this option was first presented after the birth of their baby. It emerged that some of these women after childbirth, did not feel ready to receive any postnatal care information, or to read a brochure about home-based postnatal midwifery care. Therefore, they were not ready to decide on a model of postnatal care, because they focused on other things. Two women who had given birth to their second child said:

*‘I was a little put off, I am a person who wants to please others and make things easier, so I thought we ought to say yes to that [home-based postnatal midwifery care].’* (Participant 6)

*‘It would of course have been great if I had known before, then I would probably have felt even more relaxed to be discharged.’* (Participant 12)

To receive prenatal information about postnatal models of care was not important to all the women; some said it did not play a big role or did not make any emotional difference. One mother of two children described this:

*‘For us it did not matter much, it was a nice surprise, we did not have any expectations, we knew that there is a lack of room [at the postnatal wards].’* (Participant 4)

### Time for consideration about postnatal care choices is asked for

The women described the importance of having sufficient time in making the decision about home-based postnatal midwifery care. Having had time to think and reason on whether the alternative would fit the family well, was experienced as good and smooth, and was expressed as:

*‘It was good that I did not have to decide at the minute … we [the couple] could reason together, it felt reasonable.’* (Participant 4, second child)

For others, the offer of home-based postnatal midwifery care came suddenly without time to consider, which was experienced as pressing, shocking and stressful. Therefore, some women did not understand the message and the offer of the concept home-based postnatal midwifery care. One mother of two children described this as:

*‘You are so upbeat … there is so much emotion … you are a bit in the hands of them. It was hard to know what to get too. I still think it was great. Incredibly good actually.’* (Participant 6)

Another woman did not need any time for reflection:

*‘Basically, we said yes straight up and down.’* (Participant 23, third child).

One first-time mother experienced that the caregivers tried to persuade her to decide on early hospital discharge and required a prompt answer. She said:

*‘I was not at all ready to go home because I could not stand on my feet, it felt like they wanted to get rid of us, I think they asked me four times that we had to decide.’* (Participant 9)

### The rationale for postnatal care model choice

Most of the women were free to choose between receiving postnatal care in hospital or at home and most preferred to receive postnatal care at home. The women described that it was good, nice, and safe to choose this model of care, that it suited them and that it was provided according to their wishes:

*‘I could definitely decide if I wanted to go home or not, in that way I think I have been involved in the care.’* (Participant 5, second child)

Decision on home-based postnatal midwifery care was based on different aspects such as that the childbirth was uncomplicated, that the women had previous parental experiences that they wanted to be together with their family, the positive aspects of their home environment, and the shortage of hospital beds or no reason at all.

The women described that they had to remain in the hospital for at least six hours before going home, which was appreciated for making sure that everything was ‘good’. It was valued to stay those hours because there may be questions that arise or a need for rest and sleep, before going home. Getting the option of home-based postnatal midwifery care was perceived as the midwife had made a professional assessment that everything was uncomplicated, and the women experienced confidence in the midwife’s judgement. The women felt safe in the fact that they would not have been given this option if something was not right. When the women had experienced the birth of their baby as easy, simple, fast, and uncomplicated, they felt home-based postnatal midwifery care as a good suggestion. Not having any serious problems with breastfeeding, pain, perineal trauma, micturition, fatigue and were able to move and sit down freely, were related to the fact that they felt well and thus there were no obstacle to return home from the hospital. When the women felt well, it was described as they were feeling strong and having energy, which led to them feeling safe and secure to leave the hospital. Some women had before the birth of the baby planned to return home early if everything went well with the birth. These women described at the same time an anxiety about going home with their newborn baby, if something should happen:

*‘We live quite close to the hospital, but it is still a bit, it’s not like being on a postnatal ward. I wondered if it was the right thing to do or if I should stay with my baby for her safety.’* (Participant 12, second child)

Feeling prepared for motherhood, by having previously given birth and knowing what happens after a birth, had an impact on women’s decision to go home early with midwifery care at home. This experience made the multiparous women feel prepared, be comfortable, less anxious, and rather feeling secure in the role as a mother:

*‘Everything becomes a second nature and you feel comfortable going home.’* (Participant 16, second child)

Multiparous women described the advantage of being able to be with the newborn baby’s siblings from the first day and expressed a desire to get back into normal family life as soon as possible. When some women described the advantage of home-based midwifery care from the siblings’ perspective, others described the disadvantage of returning home to the baby’s siblings early. One woman with two children said:

*‘It became very intense to come home … there was full focus on the 3-year-old, his reactions and feelings. It would have been nice if we had stayed a few more nights, I might have had time to rest up a bit.’* (Participant 10)

To be able to receive postnatal care in their own home environment was experienced as cozy and nice. Benefit of this model of care was that it minimized the risk of infection, to lie in their own bed, not to be separated from the partner and not to be left alone at a postnatal ward. Sometimes caregivers explained a lack of room at the postnatal wards, which had an impact on the women’s postnatal decision-making process; it was perceived as they wanted to get rid of the new families. One of the women said:

*‘You know how hard everyone works within maternity care … that there is a lack of space, and it affects how you choose.’* (Participant 18, second child)

Nevertheless, the women agreed that the rooms at the hospital should go to those who really needed to be in a hospital environment. For a second-time mother (Participant 10) there was no advantage seen in going home, arguing that only the caregivers benefited from it. Staying two nights at a hospital’s postnatal ward would have been desirable, with a belief in receiving more support with breastfeeding and at the same time having had time to rest a little after the birth, giving the possibility of getting into a rhythm with the new baby, before taking care of the other child.

### To comprehend the concept of postnatal care models facilitates decision-making

If all women had the knowledge of how the home-based postnatal model was organized, it would have had facilitated the decision-making process. Women would then have been more likely to assess the care as an appropriate alternative to standard care at the postnatal wards. The women considered it important, that before returning home from the hospital, to be able to put forward questions, and that verbal and written information was given about what is important to think about during the first period after the birth of the baby. The women appreciated information about the criteria for returning home from hospital and where to turn to if help was needed. Getting written information helped the women to read the information in their own pace. Many of the women described the home-based care as being appealing, and that there was no major difference between postnatal care at the hospital or at home. After receiving prenatal information about home-based postnatal care, some were critical towards the model, but after birth and having fully comprehended home-based care, it felt as the right decision.

Receiving a structured midwifery follow-up with telephone contact, home visits, and return visits to the hospital after the birth if necessary, positively influenced the women’s decision on opting for home-based midwifery care. The structured follow-up was experienced as valuable, important and security-creating, which meant that the women did not feel ‘left out’ and without contact with the midwives:

*‘It feels a bit like still being admitted [to the hospital] but still not, and I thought that was great.’* (Participant 16, third child)

Another woman expressed:

*‘It felt like we got that whole package anyway, even though we were at home.’* (Participant 4, second child).

The women appreciated and experienced a sense of security from having scheduled telephone contact made by the midwife and that they had the opportunity to call a midwife 24 hours a day. Women appreciated the phone follow-up because they could contact a midwife if they felt unsure, uncomfortable or had any questions, also if they needed a hospital appointment booked with a physician. The opportunity to receive home-visits were appreciated and one woman said:

*‘If there was anything it would be a midwife who would come home the next day so then you can collect questions until then.’* (Participant 4, second child)

## DISCUSSION

The main theme, ‘The supremacy of giving new mothers autonomy to decide on the postnatal care model they would prefer’, was explored. Important aspects of the women’s decision-making process were: the time-point for receiving information about the home-based midwifery model of care, to receive sufficient time for consideration about the model, to have a rational for choosing home-based care, and to comprehend the concept.

A postnatal care model decision should be influenced by exploring and respecting what matters most to women as individuals and this exploration in turns depends on developed informed preferences, as a mother^3,12^. However, women and their families are often excluded from reproductive decision-making processes in postnatal care, and do not know which choices they have. Shared decision-making is a critical but challenging component of maternity care quality^1,2^. If midwives are responsive to women’s preferences and needs of postnatal care, better midwifery woman-centered care will be delivered^2,3,16^. Parents and midwives in the Netherlands have given suggestions on how to improve shared decision-making in maternity care. These suggestions are to increase awareness of the model of shared decision-making, having good communication skills, and having respectful interprofessional collaboration^1,17^. Having communication skills as a midwife, and to allow women the opportunity to participate in decision-making constitute being a ‘good’ midwife according to the theory of professionalism in midwifery^18^. Women need to make reproductive decisions, often with little reflection and limited knowledge, and therefore their capacity for autonomy may be compromised^19^. The concept of autonomy is understood as the ability to ‘make and act upon free, informed decisions resulting from capable and uninfluenced deliberation’^20^. For women, self-trust in respect to care for the newborn baby is gradually acquired, and often fragile. New motherhood is inherently autonomy limiting, so postnatal reproductive autonomy should be enhanced^19^.

An important aspect, explored in our study, of the decision-making process for postnatal care model of preference, was sufficient time for consideration about different postnatal care choices before hospital discharge. To participate in the decision about time-point for postnatal discharge, the possibility of home visits and their timing have been previously described as important for women’s feelings of autonomy and control^21^. To be given enough time to consider one’s options facilitates the decision-making process thus resulting in greater autonomy and is associated with improved health outcomes^22^. According to recommendations by the Royal College of Midwives^23^, the length of hospital stay before discharge should always be discussed between each family and their midwife and be based on the health and wellbeing of the mother and baby. Because of early discharge being defined differently, it makes it difficult to evaluate the consequences of shorter length of hospital stay^24^. Early discharge has been defined as returning home within 24 hours, up to 26, 36, and 48 hours after birth^9^. Home-based postnatal midwifery care is still not the norm in Sweden^13^, despite convincing evidence that women accept and wish to be offered differentiated postnatal care^14^. Parents’ readiness for discharge must be identified by midwives, as well as they must ensure parents participation in decision-making for time of discharge^10^.

Receiving a structured midwifery follow-up influenced the women’s decision on opting for home-based postnatal midwifery care. The structured follow-up with telephone contacts, home visits, and if needed a hospital appointment, was experienced as valuable, important and security creating. According to the World Health Organization, home visits to families during the first week after childbirth have a significant reduction in the perinatal mortality rate^17^. Home-based postnatal care is also proven beneficial for women’s successful transition to motherhood, especially by helping them connect with the midwife^25^. Women have experienced a sense of security in the postnatal period when a midwife has an empowering behavior and gives new mothers informed postnatal care choices^21^ and offers a planned follow-up with consistent information^26^.

### Strengths and limitations

The qualitative research review guidelines RATS^27^ was followed. Data were collected by telephone interviews; a method considered being convenient as informants could decide for themselves the time of the interview and attend the interview without having to travel^28^. However, via telephone interviews the participants may be less cooperative than during a face-to-face interview and that body language is missed out^29^. Since the interviews were of a relatively long length and data were content-rich these issues did not appear to have negatively influenced the findings. In-depth interviews can be considered appropriate when wanting to improve or implement patient- or women-informed evidence-based maternity care^30^. To be noted, the sample size only represents women having an uncomplicated childbirth and mainly including multiparous women. The findings may therefore be transferred with caution to other groups. Although both authors have extensive experience as clinical midwives within postnatal care and in qualitative research methods, the study participants had not met or been cared for by the authors during their postnatal care period.

## CONCLUSIONS

The main theme explored was ‘The supremacy of giving new mothers autonomy to decide on the postnatal care model they would prefer’. Important aspects of the women’s decision-making process were the time-point for receiving information about the home-based midwifery model of care, receiving sufficient time for consideration about the model, having a rationale for choosing home-based care and comprehending the concept. Parents’ readiness for discharge must be identified by midwives who need to facilitate shared decision-making by introducing early postnatal care model choices, describing the options, and supporting women to explore their preferences. Midwives must ensure parents participation in decision-making for the postnatal care model.

## Data Availability

The data supporting this research are available from the authors on reasonable request.
